# Association between food insecurity and cardiometabolic risk in adults and the elderly: A systematic review

**DOI:** 10.7189/jogh.10.020402

**Published:** 2020-12

**Authors:** Elizangela da Silva Miguel, Sílvia Oliveira Lopes, Susilane Pereira Araújo, Silvia Eloiza Priore, Rita de Cássia Gonçalves Alfenas, Helen Hermana Miranda Hermsdorff

**Affiliations:** Department of Nutrition and Health, Universidade Federal de Viçosa, Viçosa-Minas Gerais, Brazil

## Abstract

**Background:**

Food insecurity is a public health concern that affects health and quality of life, but its association with cardiometabolic risk is not well established. Thus, this systematic review evaluated the association between food insecurity and cardiometabolic risk factors in adults and the elderly.

**Methods:**

Search was conducted according to the PRISMA protocol using Scielo, LILACS and PubMed databases. We included original articles published in Portuguese, English, and Spanish, which assessed the association between food insecurity and cardiometabolic risk factors in adults and the elderly. The search identified 877 articles but only 11 were included in the review.

**Results:**

Food insecurity was directly associated with cardiometabolic risk (excess weight, hypertension, dyslipidemias, diabetes, and stress) after adjusting for interfering factors. A limitation of the cross-sectional study design is that the cause-effect relation between food insecurity and cardiometabolic risk cannot be established.

**Conclusions:**

We conclude that food insecurity has a direct relationship with cardiometabolic risk factors, especially excess weight, hypertension, and dyslipidemias. The identification of food insecurity as health problems can contribute to the implementation of efficient public policies for the prevention and control of chronic diseases.

**Protocol registration:**

This review was registered on PROSPERO-International Prospective Register of Systematic Reviews – CRD4201911549.

Food security is defined as the physical and economic access to safe and nutritious food that meets individual and household needs for the promotion and maintenance of health. Failure to satisfy the Human Right to Adequate Food and Food Sovereignty results in food insecurity. In 2017, 10.9% of the world's population was food insecure and concomitantly, the prevalence of obesity increased from 11.7% in 2012 to 13.2% in 2016 [1].

A direct measure of food insecurity through perception was first developed by researchers of Cornell University [2]. This method was later applied in the US census [3]. The Household Food Security Survey of the US Department of Agriculture (USDA) measures the possibility of running out of food before affording to buy more. This tool also considers severe food deprivation, where adults and children go all day without eating [4]. Food insecurity can be measured indirectly through indicators such as anthropometry [5], food consumption [6], food expenditure [7] and imposition of food patterns that do not respect cultural diversity [8].

Food insecurity has significant impact on nutritional status, food consumption, and food access. Furthermore, studies have shown that food insecure individuals tend to consume less whole grains and more refined carbohydrates and sodium, increasing the risk of morbidity and mortality [9,10]. Thus, food insecurity could be related to risk of developing chronic cardiometabolic diseases, such as obesity, diabetes, hypertension, and dyslipidemias [9,11-13].

Overall, the present systematic review aimed to evaluate the association between food insecurity and cardiometabolic risk factors in adults and the elderly.

## METHODOLOGY

The guiding question employed was “Is food insecurity directly associated with cardiometabolic risk factors in adults and the elderly?”. The question is based on increasing prevalence of food insecurity worldwide at the same time that chronic diseases such as obesity and related cardiometabolic risk factors [1].

### Protocol and registration

This systematic review was conducted according to the PRISMA protocol-Preferred Reporting Items for Systematic Reviews and Meta-Analysis [14] and was registered on PROSPERO-International Prospective Register of Systematic Reviews-CRD42019115493.

### Inclusion criteria

We included original articles published in Portuguese, English, and Spanish, which assessed the association between food insecurity and cardiometabolic risk factors in adults and the elderly. Only articles indexed in Scielo, LILACS and PubMed databases were considered. Also, no restriction was placed on the year of publication. The references of the articles were searched, and other articles not identified in the initial search were included (reverse citation search).

### Search, selection and evaluation of articles

**Figure 1:**
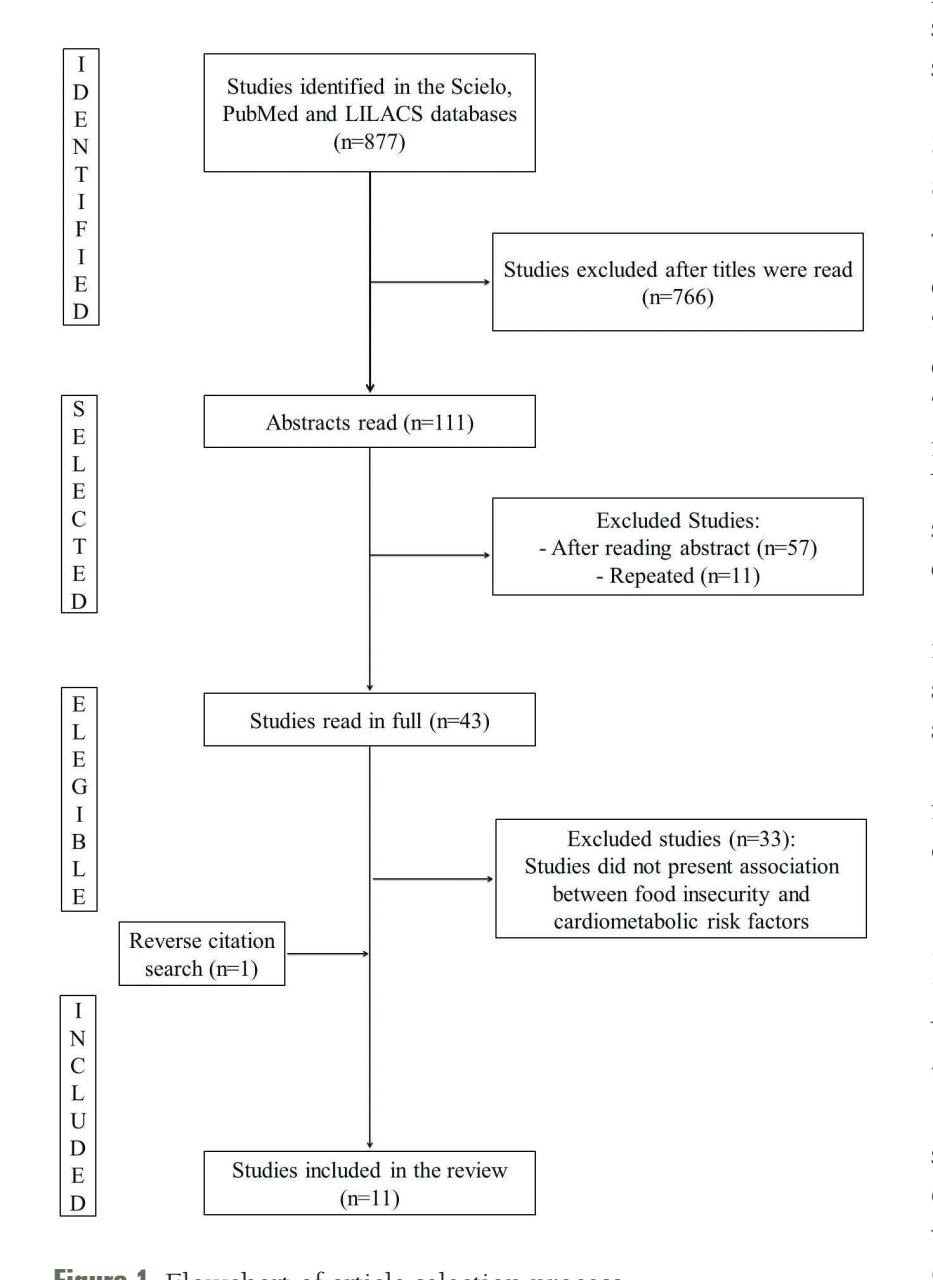
Flowchart of article selection process.

The search terms used were 'food security', 'food insecurity', 'diabetes', 'hypertension', 'metabolic syndrome', 'dyslipidemia', 'stress', 'waist circumference' and ' excess weight'. For the search, we used a combination of 'food security' OR ‘food insecurity’ AND other terms, to refine our search to the topic of interest, the relationship between food insecurity and cardiometabolic risk. The search produced 877 articles, however 766 were excluded after reading the titles. The abstracts of the remaining 111 articles were read. Then, 43 studies were selected for further reading. After reading the articles in full, 10 articles were selected. An additional article was selected after a reverse citation search, totaling 11 articles (**Figure 1**). The studies were evaluated by STROBE - Strengthening the Reporting of Observational Studies in Epidemiology [15].

## RESULTS

We identified 11 cross-sectional studies published between 2006 and 2017, conducted in the United States [16-24], Mexico [25] and Malaysia [26]. In relation to study population, 54.5% (n = 6) of the studies were conducted with adults only [17-19,21,24,26], 36.4% (n = 4) with both adults and the elderly [16,20,22,23], and 9.1% (n = 1) with only the elderly [25].

The majority of the studies, 64% (n = 7), assessed food insecurity by the Household Food Security Survey (USDA) [16-19,22,24,26]. However, the studies presented a wide range of food insecurity assessment tools (**Table 1**).

**Table 1:** Stratification of food insecurity according to the reviewed studies

Author (year)	Stratification
Holben, Pheley (2006) [16]	−*
Seligman et al (2007) [17]	FS – No affirmative answer
	Mild FI – 1 to 5 affirmative answers
	Severe FI – 6 to 10 affirmative answers
Jilcott et al (2011) [18]; Berkowitz et al (2013; 2017) [19,24]	FS – 0 to 2 affirmative answers
	FI – 3 or more affirmative answers
Pérez-Escamilla et al (2014) [25]	FS – 0
	Mild FI – 1 to 3 points
	Moderate FI – 4 to 6 points
	Severe FI – 7 to 8 points
Shariff et al (2014) [26]	FS – 0
	FI – 1 to 8 points
Irving, Njai, Siegel (2014) [20]	FS – Answer was rarely or never
	FI – Answer was always, usually or sometimes
Moreno et al (2015) [22]	FS – 0 to 1 affirmative answer
	FI – 2 affirmative answers
Shin et al (2015) [21]; Saiz Júnior et al (2016) [23]	An affirmative response to any of the FI assessment questions

FS – food security, FI – food insecurity

*Stratification method was not disclosed.

**Figure 2:**
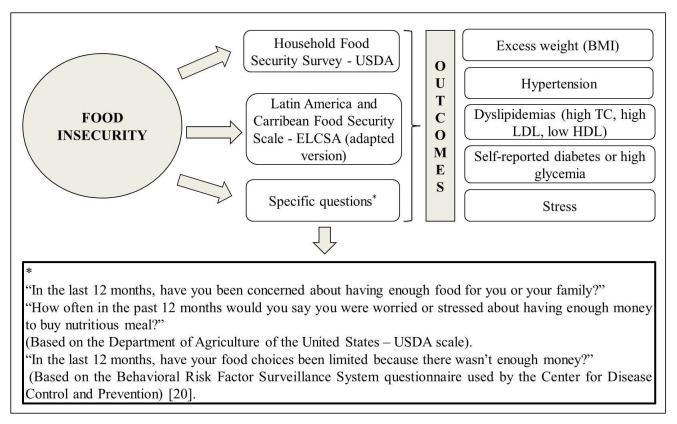
Graphical representation of the instruments used to evaluate food security and clinical outcomes in studies with adults and the elderly, 2018. BMI – body mass index, TC – total cholesterol, HDL – high density lipoprotein, LDL – low density lipoprotein

The studies evaluated the cardiometabolic risk through body weight excess according to the body mass index (BMI), high blood pressure (BP), high total cholesterol (TC), high LDL, low HDL, presence of diabetes or stress. In 73% (n = 8) of the articles, cardiometabolic risk was assessed by excess body weight and 64% (n = 7) by high BP. Food insecurity was directly associated with all evaluated cardiometabolic risk factors evaluated (**Figure 2**). The associations were maintained after adjusting for sociodemographic, economic and lifestyle characteristics. In contrast, three studies showed food insecurity was inversely associated with cardiometabolic risk factors, specifically, increased BP and LDL, metabolic syndrome and obesity in women. ^22; 23; 26^ The detailed results of the studies are described in **Table 2**.

**Table 2:** Evaluation of the association between food insecurity and cardiometabolic risk in adults and the elderly

Author/Year	Place/Sample	Evaluation method		Association between FI and CRM
**FI**	**CRM**
Holben, Pheley (2006) [16]	United States, n = 2580	Household Food Security Survey – USDA	-Excess weight (BMI)	Obesity:>prevalence among insecure individuals
			-High diastolic BP	FI: Directly associated with o excess weight (BMI) in women
			-High TC	
			-Self-reported Diabetes	
Seligman et al (2007) [17]	United States, n = 4423	Household Food Security Survey – USDA	-Excess weight (BMI)	Women with FI women:>occurrence of obesity in secure women or severe FI
			-Self-reported diabetes	Participants with severe FI>probability of having diabetes than food secure participants, after adjusting for socio-demographic factors, level of physical activity and BMI
			-High WC	
Jilcott et al (2011) [18]	United States, n = 202	Household Food Security Survey – USDA	-Excess weight (BMI)	FI: Directly associated with excess weight (BMI) and perceived stress
			-Stress	
Berkowitz et al (2013) [19]	United States, n = 2557	Household Food Security Survey – USDA	-High BP	FI: Directly associated with high glycemia and cholesterolemia, after adjusting for sociodemographic factors, smoking, BMI, diabetes duration, statin use and being under treatment.
			-High cholesterol	
			-Diabetes	
Irving, Njai, Siegel (2014) [20]	United States, n = 58 677	Question:	-High BP	FI: directly associated with hypertension, after adjusting for age, sex, race/ethnicity, level of education and poverty, health insurance coverage, marital status, and smoking.
		“How often in the past 12 months would you say you were worried or stressed about having enough money to buy nutritious meals?”		
Pérez-Escamilla et al (2014) [25]	Mexico, n = 32 320	Adopted version of the Latin America and Caribbean Food Security Scale	-High BP	FI mild, moderate and severe: directly associated with the presence of diabetes and hypertension in women
			-Diabetes	
Shariff et al (2014) [26]	Malaysia, n = 625	Household Food Security Survey – USDA	-Excess weight (BMI)	FI: Inversely associated with increased LDL, metabolic syndrome and obesity in women
			-High BP	
			-High TC, low HDL, high LDL	
			-High WC	
			-Metabolic syndrome (presence of 3 or more factors)	
Moreno et al (2015) [22]	United States, n = 250	Household Food Security Survey – USDA	-Excess weight (BMI)	FI: associated with BP and LDL
			-High BP	
			-High LDL	
			-Diabetes	
Shin et al (2015) [21]	United States, n = 1663	Questions	-Excess weight (BMI)	FI: Associated directly with low HDL among women, after adjusting for age, race, level of education, family income, smoking, alcohol intake and physical activity
		-“In the last 12 months, have you been concerned about having enough food for you or your family?”		
		-“In the last 12 months, have your food choices been limited because there wasn’t enough money?”	-Dyslipidemia (high TC or low HDL)	
Saiz Júnior et al (2016) [23]	United States, n = 2935	Question:	-Excess weight (BMI)	FI: Inversely associated with hypertension, TC and BMI
		- “In the last 12 months, have you been concerned about having enough food for you or your family?”	-High BP	
			-High TC	
Berkowitz et al (2017) [24]	United States, n = 21 196	Household Food Security Survey – USDA	-Excess weight (BMI)	FI: directly associated with hypertension, diabetes, obesity, and LDL
			-High BP	
			-High LDL	
			-Diabetes	

FI – food insecurity, CRM – cardiometabolic risk markers, USDA – US Department of Agriculture, BMI – body mass index, BP – blood pressure, TC – total cholesterol, HDL – high density lipoprotein, LDL – low density lipoprotein, WC – waist circumference, ELCSA – Latin America and Caribbean Food Security Scale

The selected articles presented a median score of 18 points as regards quality (minimum = 14, maximum = 21).

## DISCUSSION

Most of the studies presented association between food insecurity and cardiometabolic risk factors in adults and the elderly, regardless of interfering factors. In this context, some relevant issues can be discussed.

First, studies on the physical and economic access to food should not focus solely on identifying the absence of food but rather the inadequate access to the same, which may be related to chronic clinical outcomes. In fact, the analysis of socioeconomic factors can elucidate clinical outcomes since its deprivation can promote inadequate food consumption [27,28]. The consumption of high calorie foods has been shown to increase the risk of diabetes and arterial hypertension [29,30].

On the other hand, prolonged food deprivation may cause an inverse association between food insecurity and cardiometabolic risk factors because individuals in this situation lack food diversity and tend to reduce their portion size [31-33]. In turn, medical expenses related to cardiometabolic diseases can restrict the quantity and quality of healthy food consumed, which in turn increases the risk of food insecurity, when resources are limited. Accordingly, food insecurity and cardiometabolic risk can form a vicious cycle [34]. Moreover, stress increases cortisol concentrations, contributing to an increase in adiposity, possibly leading to obesity and altered blood pressure [29,30]. It is important to mention that similar results have been found in children and adolescents.

The second point throws light on the instruments used for the assessment of food insecurity. Food insecurity can be measured by household food availability, dietary intake, anthropometric, socioeconomic, biochemical and clinical data. However, food insecurity scales based on perception are mostly utilized because they allow a direct classification and stratification of severity (mild, moderate, and severe) [35,36]. The American scale, considered “gold standard”, and the Latin America and Caribbean Food Security Scale, validated for the Mexican population, are instruments which provide information on the distribution, causes and consequences of food insecurity, most importantly, the magnitude of the same based on reliable and validated indicators [37].

In addition, associations established depend on the stratification and psychometric analyses of the answers and guiding questions. Furthermore, scales are direct indicators of food insecurity, thus can be used as monitoring tools. They also facilitate the identification of vulnerable groups [37]. However, scales only measure the perception of insecurity, leaving out the nutritional aspect of food insecurity.

In this sense, the evaluation of food insecurity considering the nutritional status of individuals could clarify controversial values reported in some studies [33,37,38]. On the other hand, scales predict household food insecurity than individual food insecurity. In this sense, future studies should focus on the measurement of individual food insecurity based on the stratification of socioeconomic factors since they can be contributing factors.

Regarding anthropometric markers used in the studies, we highlight BMI and waist circumference as simple, fast and inexpensive methods for assessing cardiometabolic risk. Moreover, these markers have a good association with adiposity.

We would like to discuss about age-groups distribution of the study samples. In fact, we adopted as selection criterion to search studies with adults and, or, elderly, based on the lack of global information addressing the relationship between food insecurity and cardiometabolic risk in these age-groups as well as on the hypothesis this would be more prevalent among older persons. In this sense, we found greater number of studies with adults, followed by studies with two age-groups and studies participating only elderly. However, the study outcomes were similar, with positive association between food insecurity and cardiometabolic risk factors, regardless of age sample. Our review indicates food insecurity as relevant factor in these two age-groups on developing chronic diseases, at least at the moment.

The strengths of this review are the use of secondary data from representative samples, and the measurement of food insecurity using “gold standard” tool, in its original or adapted version, by included studies. As limitation, all the included studies had cross-sectional design, so the cause-effect relation between food insecurity and cardiometabolic risk cannot be established. In addition, the scales do not allow an assessment of the nutritional aspect and the assessment of the situation of food insecurity is at the household level, while the cardiometabolic risk factors were assessed at the individual level. Therefore, longitudinal studies in humans that consider the nutritional dimension and the individual aspect would allow establishing a causal relationship between food insecurity and cardiometabolic risk.

## CONCLUSIONS

Food insecurity has a direct relationship with cardiometabolic risk factors, especially excess weight, hypertension, and dyslipidemias, indicating the need to assess individual and household food insecurity. The identification of food insecurity as health problems can contribute to the implementation of efficient public policies for the prevention and control of chronic diseases.
